# Learning Priors for Bayesian Computations in the Nervous System

**DOI:** 10.1371/journal.pone.0012686

**Published:** 2010-09-10

**Authors:** Max Berniker, Martin Voss, Konrad Kording

**Affiliations:** 1 Department of Physical Medicine and Rehabilitation, Northwestern University and Rehabilitation Institute of Chicago, Chicago, Illinois, United States of America; 2 Department of Psychiatry and Psychotherapy, Charité University Hospital & St. Hedwig Hospital, Berlin, Germany; Mount Sinai School of Medicine, United States of America

## Abstract

Our nervous system continuously combines new information from our senses with information it has acquired throughout life. Numerous studies have found that human subjects manage this by integrating their observations with their previous experience (priors) in a way that is close to the statistical optimum. However, little is known about the way the nervous system acquires or learns priors. Here we present results from experiments where the underlying distribution of target locations in an estimation task was switched, manipulating the prior subjects should use. Our experimental design allowed us to measure a subject's evolving prior while they learned. We confirm that through extensive practice subjects learn the correct prior for the task. We found that subjects can rapidly learn the mean of a new prior while the variance is learned more slowly and with a variable learning rate. In addition, we found that a Bayesian inference model could predict the time course of the observed learning while offering an intuitive explanation for the findings. The evidence suggests the nervous system continuously updates its priors to enable efficient behavior.

## Introduction

For any sensorimotor behavior, we rely on our sensory inputs as well as knowledge we have accumulated over the course of our life. For example, when descending stairs we use our visually perceived assessment of stair width and depth, but also our experience of these typical attributes (as is evident when we descend stairs without looking). Bayesian statistics provides a way of calculating how prior knowledge can be combined with new information from our senses in a statistically optimal way. A wide range of studies has found that human behavior is close to these predictions of optimal combination. In particular, the Bayesian use of prior information has been observed in tasks such as the perception of visual movement [1], [2], cue combination [3], [4], visuomotor integration [5], [6] movement planning [7] and motor adaptation [8]. While these studies have shown that human subjects can efficiently use prior information, little is known about the way such priors are learned.

**Figure 1:**
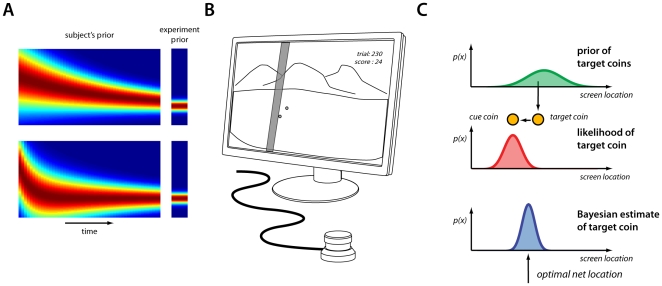
Illustration of potential learning patterns and overview of experiment. A) Possible depiction of the subject's estimated prior while they learn both mean and the variance at the same rate (top), or when the learning rate for the variance is slower (bottom), as would be expected from statistical considerations. B) A depiction of the experimental set-up. Coins are displayed on screen and the subjects place a horizontal “net” with a paddle wheel. C) A distribution over coin locations defines the prior, the observed cue coin defines a likelihood for the target coin, and Baye's rule prescribes the optimal posterior distribution of the estimated location of the target coin.

Based on experimental evidence (e.g., [9], [10]) it is clear priors can change, however, it is unclear *how* these priors change over time, if they converge to the veridical distribution, and over what time scales they change. We began our study with two hypotheses concerning learning rates. It could be the case that both the mean and the variance of a prior are learned at the same rate (see Fig. 1A), perhaps mediated by the same neural mechanisms. Consistent with many computational models of learning (e.g., [11], [12]) neural processes of adaptation could fix this rate. Alternatively, learning the mean and variance may proceed at different rates (Fig. 1A) possibly mediated by distinct neural mechanisms. This would be consistent with statistical considerations if the different variables have differing levels of uncertainty. Similarly, recent evidence suggesting specific cortical regions modulate learning rates [13] based on representations of uncertainty [14], [15] implies this type of learning may be the norm. Our aim was to determine how human subjects learned a prior and what strategy may have been responsible for it.

We designed three versions of a “coin catching” experiment to examine if subjects could not only estimate a prior, but also independently estimate both the mean and the variance of the prior. In the first experiment we found evidence that subjects eventually estimated an accurate prior, and the number of trials necessary for this. In the second experiment we found that subjects could learn the variance of a prior independent of their estimate of the mean. In the third experiment we found evidence that subjects could correctly estimate multiple means for a prior, and when to switch between them. For comparison we examined multiple Bayesian inference models and compared their performance on the same tasks. This analysis offered an intuitive explanation for the seeming changes in learning rates subjects exhibited as the experiment progressed. In addition, we found that subjects were able to infer a new mean in the prior nearly as quickly as a Bayesian inference model. Taken together, we present strong evidence that subjects are capable of acquiring an accurate prior, with multiple and variable learning rates, and using it to make statistically optimal decisions.

## Methods

In a “coin catching” task we investigated how subjects adapted their expectation of coin locations (a prior) in response to changes in the underlying distribution. Subjects had to estimate the location of a virtual target coin, randomly drawn from a normal distribution. On every trial, subjects were given noisy information of the coins current location, in the form of a single “cue coin” (similar to the experimental design of [10]) and were then asked to guess the location of the “target coin”. To successfully estimate the target coin's location, subjects needed to integrate the coin's likelihood (obtained from the cue coin) with its prior (the distribution of previous target coin locations). By collecting data on where the subjects estimated the target coin to be, we could then estimate the prior used by the subject and analyze its temporal evolution.

### Experimental protocol

All experimental protocols were approved by the Northwestern University Institutional Review Board and were in accordance with Northwestern University's policy statement on the use of humans in experiments. All participants were naïve to the goals of the experiment, signed consent forms and were paid to participate. Subjects were seated in front of a computer screen and given an electronic paddle wheel (Griffin PowerMate). On a computer monitor a thin vertical bar the height of the screen was superimposed above a natural lake setting. Subjects were shown how to use the paddle wheel to control the location of the “net” (the thin bar) on the screen. A program created specifically for these experiments (in Matlab) obtained the positions of the target coin from a specified Gaussian distribution. This distribution was the experiment's prior. The target coin's horizontal location was then used as the mean of a second Gaussian distribution to randomly draw the location of the cue coin. In each trial, this cue coin was displayed first and only extinguished at the trials conclusion. The subjects would then move the net to the location they believed would most likely “catch” the target coin. Since the net covered the entire height of the screen only the horizontal location was relevant, rendering this a one-dimensional task. After the paddle wheel was depressed the target coin was displayed, with an indication of whether or not the subject had successfully “caught” it and what their running score was (Fig. 1B). Thus the cue coin was noisy evidence of the target coin's location: the likelihood. The mean of this distribution was defined by the location of the cue coin. Similarly, the variance of this distribution was observed from trial to trial through the spread between cue and target coin locations. To correctly estimate the target coin's location, subjects would have to integrate this likelihood with an estimated prior over target coin locations. By remembering the target coin's location from trial to trial, subjects can infer this prior.

With slight variations (see below) subjects were instructed that an individual standing behind them was tossing coins, two at a time. These two coins left the individual's hand at the same time, and flew towards the lake. The first coin the subjects saw (the cue coin) landed in the lake first, and they were to place the net where they were most likely to catch the second (target) coin, currently in mid-flight and about to land. They could take as much time as they needed. Once they had appropriately placed the net and depressed the paddle wheel they would find out if they caught the second coin. Additionally, they were instructed that the individual was neither trying to help them nor prevent them from catching the coins, and would not change the way he tossed the coins in response to their behavior.

The target coin was caught if the net and the target coin overlapped by at least 50% of the coins width. The screen units were normalized between −0.5 (the left edge) and 0.5 (the right edge). In each experiment the standard deviation of the likelihood distribution's was fixed at 0.1 (one tenth of the screen's width). As we were interested in the learning of priors, only their distributions were manipulated (Fig. 1C). To ensure each subject inferred a unique prior, a different random location centered at the middle of the screen was used to define the prior's mean for each subject (drawn from a normal distribution with zero mean and a standard deviation of 0.1). In the first experiment the prior's variance was one of two values, either 0.05 or 0.2. All subjects in experiment one were given the instructions described above and asked to perform 400 trials.

In the second experiment subjects were given identical instruction. Two values were used for the variance, the same two values as used in experiment one (0.05 and 0.2). One of these two values was randomly chosen at the start of the experiment and assigned to the prior. After half of the trials (250) the prior's variance would switch to the other value. All subjects performed 500 trials in total after receiving the instructions described above.

In the third experiment two locations were used for the prior's mean, −0.05 and 0.05. After the completion of 10 trials, the prior's mean would flip to the other location based on a Bernoulli probability of (p = 0.2). The prior's variance was 0.1 throughout the experiment. Subjects were given the instructions described above, however, they were given the additional information that at random times, the individual tossing the coins would move. All subjects were asked to perform 600 Trials.

### Data analysis

In order to perform the coin catching task successfully, in each trial the net should be placed in the most likely location of the target coin. According to Bayes' rule, this is given as, μ_t_ = (1−r) μ_p_+rμ_l_, where μ_p_ and μ_l_ are the mean of the prior, and the mean of the likelihood (the cue coin's location) and μ_t_ is the mean of the target coin's probability. *r* is defined through the variances of the prior and likelihood (*r* = *σ_p_^2^*/(*σ_p_^2^*+*σ_l_^2^*)). In effect, *r* is an alternate form of the Kalman gain. By recording the cue coin's location, and assuming the subjects placed the net where they believed the target coin to be (μ_t_), we can infer the parameter, *r*, and their belief of the prior's mean.

For all experiments, we wanted to examine both what subjects learned (estimates of *r* and μ_p_), as well as how these parameters progressed across time. Therefore, for all subjects, each consecutive ten trials were binned together. This binned data was then used to fit *r* and μ_p_ (in a linear least squares sense). Through this procedure we obtained measures of how the subject's estimates of the prior developed in time. For experiment 3, we resorted the data aligned to a switch in the prior's mean and then binned all the data in the subsequent 10 trials. Thus we could infer each subject's estimate of the prior's mean, after a change in its value.

### Bayesian inference analysis

For all the experiments, subjects would need to estimate the prior's mean and variance to successfully complete the trials. In experiments 1 and 2, we wanted to examine how subjects adapted their estimate of both the mean and the variance of the experimental prior. Therefore, we designed a Bayesian inference model to perform the same experiments (through simulation) and to estimate both the mean and the variance of the experimental prior. To simplify the analysis and make the model further accurate, we assumed it accurately knew the likelihood's variance. The model's performance was then used as a benchmark to compare with the subjects. In experiment 3 every subject was exposed to each of the two means for approximately 50 blocks of trials during the course of the 600 trial experiments. Furthermore, from the results of experiments 1 and 2 we concluded that subject's could estimate the prior's mean relatively quickly. Based on this we designed a Bayesian inference model that knew the prior's correct variance, but had to infer which of the two means was currently being used. This model's results were also compared with subject behavior.

To analyze experiments 1 and 2 with our Bayesian approach we need to propose probabilistic description of the prior's mean and variance. If the mean and variance were continuous and unbounded variables then they could be represented with Gaussian distributions and a Kalman filter could be used to optimally estimate them. The prior's mean can easily be modeled in this manner. The variance, however, is only defined for positive values and cannot be accurately described this way. Instead we must limit ourselves to an appropriate non-negative distribution. To do this we use the Normal-scaled Inverse Gamma (*NIG*) distribution (see Appendix S1). There are multiple advantages to using this representation. The *NIG* distribution is a conjugate prior for a Gaussian likelihood, so we can easily update its parameter values to compute a posterior distribution. Further, it correctly limits the variance to non-negative values, and assigns vanishingly small probabilities to a variance of zero.

For experiments 1 and 2 we used the *NIG* distribution to examine the performance of a Bayesian inference model. The model has four free parameters. For a given set of these parameters, we could simulate many experiments to find the model's average estimates for, μ_p_ and *σ_p_^2^* and the resulting *r*. To obtain a ‘best’ set of the four free parameters and avoid over-fitting, we found the values that maximized the log likelihood of observing the across subject average binned data, means, μ_p_ and gains, *r*, from experiment's 1A and 1B. With these parameter values fixed, the Bayesian inference model was then used to simulate many runs of experiment 2 to find the model's average performance. See Appendix S1 for more details. These results could then be compared with the across subject average performance. Finally, as a further point for comparison, we also examined a linear model that only estimated the prior's variance. This model used a running window of the last ten trials to compute a sample observation of the prior's variance. The model then used this observation to update its estimate of the variance using a fixed learning rate; the model is essentially a linear filter (see Appendix S1). Using these two models as a point of reference, we could compare subjects' performance with both a simple model, and a Bayes' optimal model of inference.

In experiment 3, during the course of the 600 trials of this experiment, each subject was exposed to each of the two means for approximately 50 blocks of trials. Based on this, and similar considerations noted above, we assumed the inference model had access to the true variance but had to infer which of the two means was currently being used. For this analysis, the inference model used the coins displayed on each trial to compute the probability that either mean was currently in use, and the most likely target location. In contrast with the previous Bayesian inference model, there were no free parameters. As with the analysis of experiments 1 and 2, we simulated many identical versions of experiment 3 (each with 600 trials) to obtain the average model performance. See Appendix S1 for more details.

## Results

We designed an experiment where we monitor a subject's prior while they take part in a simple computerized game. In this game, subjects are asked to try and catch coins with a net. On every trial the subject is presented with noisy evidence of the coin's location (a cue coin). The subject then places a virtual net where they think they are most likely to catch target coin. Subjects were instructed to attempt to catch as many coins as possible. The locations of the cue coin were drawn from a distribution centered on the target coin. Thus the cue coin by itself provides the subject with evidence of where the target coin will land, defining a likelihood. The variance of this likelihood was held constant throughout all experiments. The mean of this likelihood however, was drawn from a precursor distribution, the prior. Therefore, to accurately predict the location of the target coin on each trial, subjects would need to estimate the prior and integrate this information with the likelihood. In experiment 1 the prior was held constant, in experiment 2 the variance of the prior changes and in a third experiment the mean of the prior was changed periodically. As a result, we could assess if subjects could estimate a prior, and if so, whether they independently learned both the variance and mean of this prior.

### Experiment 1

**Figure 2:**
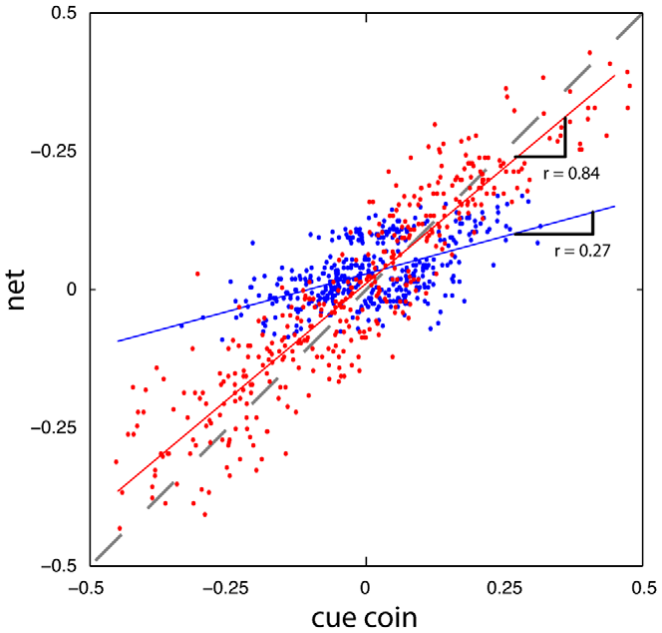
Data from two representative subjects in experiment 1. For illustrative purposes, the mean of the target coin's prior was removed from the cue coin's positions to center the data. For the subject in group 1A (the red dots), on average the net was placed relatively close to the cue coin (the diagonal represents net  =  cue coin). For the subject in group 1B (the blue dots), on average the net was placed relatively close to the mean of the target coins (far from the diagonal). Linear fits to all 400 trials of the experiment are shown in the solid red and blue lines. The Bayes' optimal slopes are 0.8 and 0.2.

For each subject, one of two values, a large or small value, was used to define the variance for that experiment's prior. The mean for the prior was chosen from a random location on the screen at the start of each experiment. We found that given enough trials, subjects tended to acquire a strategy that reflected an accurate prior. For subjects exposed to the large variance (group 1A, N = 7), cue and target coins appeared over relatively broad regions of the screen. These subjects tended to choose net locations relatively close to the displayed cue coin, as Bayesian integration would prescribe (see Fig. 2). Similarly, for subjects exposed to the small variance (group 1B, N = 7), cue and target coins appeared in a relatively narrow region of the screen. These subjects tended to choose net locations relatively close to the mean of the prior, again consistent with Bayesian integration (see Fig. 2).

**Figure 3:**
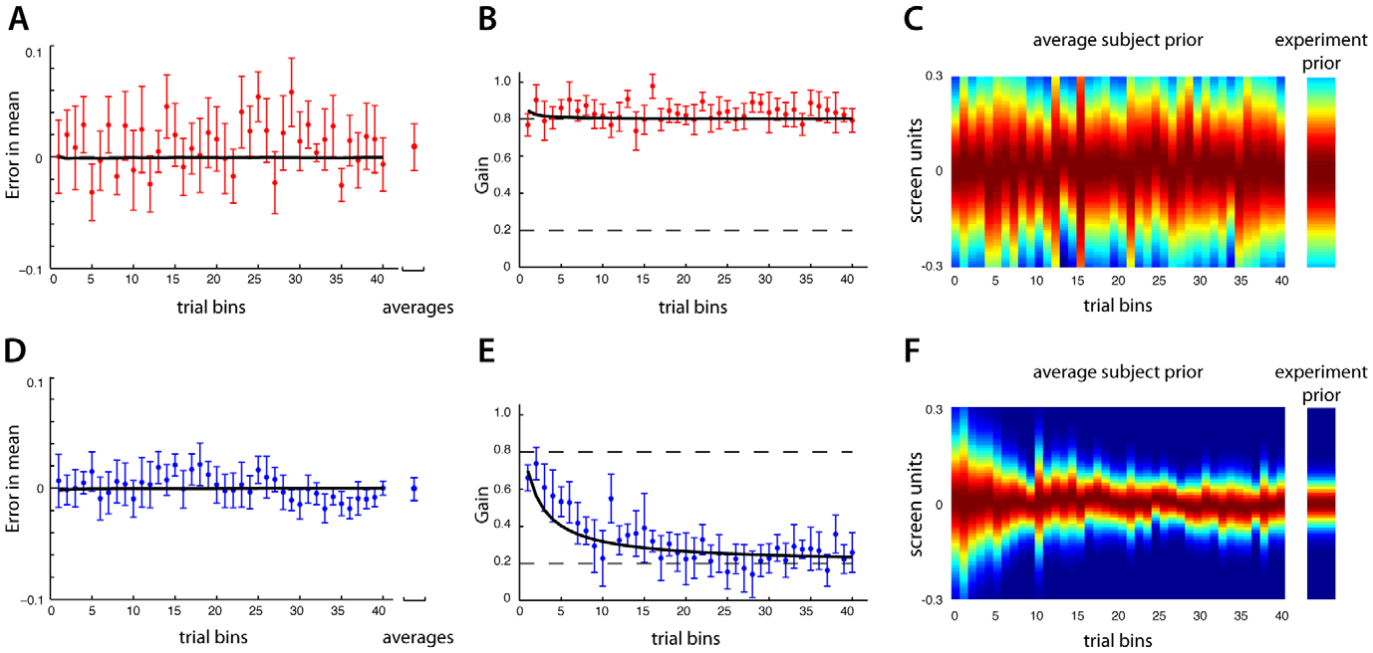
Results from experiment 1. Group 1A's results (large variance group) are in the top row and group 1B's results (small variance group) are in the bottom row. A), D) The error in the estimated mean of the prior, over the course of the experiment (each bin is 10 consecutive trials) averaged over subjects (mean +/− standard error). The far right point is the average over subjects and trials. B), E) The gain, *r*, subjects used during the experiment, averaged across subjects. The bold black line indicates the Bayesian inference model's fits to the experimental data. C), F) Inferred average prior as it evolved over the experiment.

We estimated the mean for each subject's prior (see Methods) using bins of 10 consecutive trials. According to our analysis, on average subjects quickly learned the mean of the experimental prior, nearly within the first 10 trials. This was true for both the large and small variance groups (see Fig. 3A, D). However, as would be expected based on statistical principles (the variance of a sample mean is proportional to the distribution's standard deviation), individual subjects were less accurate in estimating the mean under the large variance condition. This can be observed qualitatively by the larger variability in their estimates (compare standard error bars, Fig. 3A, D). The variability of group 1A was significantly greater than group 1B (two sample *t*-test, *p<0.001*). Nonetheless, both groups maintained estimates that were similar to the correct values.

In contrast with the fast acquisition of the prior's mean, we found that subjects could require many trials to converge on an accurate estimate of the variance. To infer each subject's estimate of this variance we measured the relative weighting subject's placed on the cue coin relative to the prior's mean, when estimating the target coin's location (the slope in Fig. 2). This gain, *r* is a measure of the subject's estimate of the prior (see Methods). If subjects believed the prior had a wide distribution, *r* would be close to 1.0, indicating the cue coin was the best proxy for the target coin's location. Similarly, if the subject's believed the prior had a narrow distribution, the gain *r*, would be close to 0, indicating the prior's mean was the best proxy for the target coin. We computed this gain over bins of 10 consecutive trials. Across all subjects, *r* took on relatively large values in the first trials, indicating the subject's belief in a “flat” prior; e.g. the subject's displayed little preference for initially believing the coins would appear in any expected location. However, as the experiment progressed *r* converged to the correct value. For group 1B, the data averaged across subjects indicated that approximately 200 trials (20 bins) were required to correctly estimate the variance of the prior (see Fig. 3E). Considering only the last 50 trials, we found that the across-subject average *r* value was not significantly different from the true value (two sample *t*-test, *p>0.1*). Due to the subjects' predisposition for a flat prior, subjects in group 1A essentially began the experiment with the correct gain (see Fig. 3B, the last 50 trials were also not significantly distinct from the true value, *p>0.1*). Using the values inferred for the mean and variance, we were able to reconstruct the subject's estimated prior during the course of learning (Fig. 3B, E). This analysis shows that human subjects converge to the correct variance of the prior with a timescale on the order of a hundred trials.

### Experiment 2

**Figure 4:**
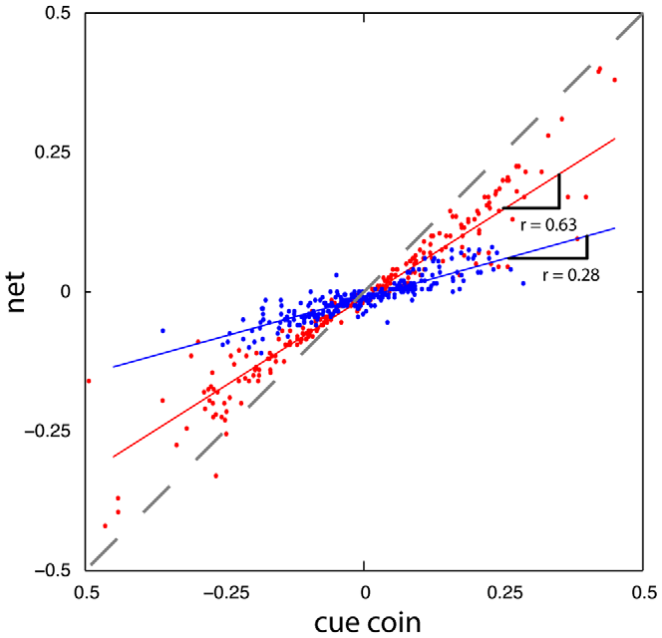
Data from a representative subject in experiment 2B. For illustrative purposes, the mean of the target coin's prior was removed from the cue coin's positions to center the data. During the first half of the experiment the subject was exposed to a prior with a narrow variance (blue dots), and on average, the subject placed the net relatively close to the target coin's mean location (far from the diagonal). In the second half of the experiment the prior's variance was wide and the subject placed the net progressively closer to the cue coin (the red dots). Linear fits to the first 250 trials (blue line) and the last 250 trials (red line) are shown. The Bayes' optimal slopes are 0.8 and 0.2.

From the results of experiment 1 we concluded that subjects could learn to properly incorporate a prior into their strategy, and the approximate number of trials that were required. However, it could be that subjects didn't learn to correctly represent the prior, but rather developed a heuristic that performed the same function. Therefore, we wanted to assess whether the individual parameters of the prior's distribution were accurately estimated. The second experiment was thus designed to examine subjects' ability to learn the prior's variance. One of the two variances used in experiment 1 were randomly chosen to begin the experiment. After half of the trials (250) the prior's variance would switch to the other value. Only a single switch during this experiment was performed since experiment 1 indicated that learning the variance took many trials (∼200). In all, 14 subjects performed experiment 2, completing 500 trials. Seven subjects were first exposed to the large variance (group 2A), and seven subjects were first exposed to the small variance (group 2B, see Fig. 4). In order to successfully complete the task, each subject would have to infer when the variance switched, and what this new value was.

**Figure 5:**
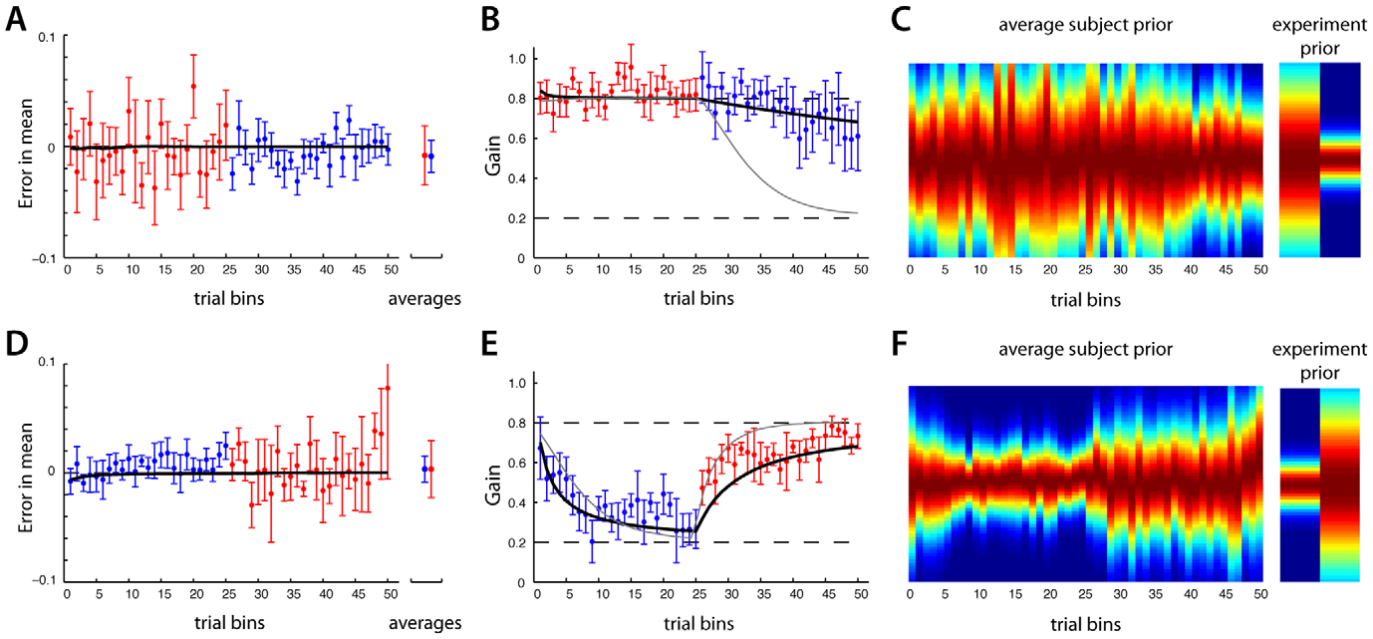
Results from group 2. Group 2A's results (large variance first) are in the top row and group 2B's results (small variance first) are in the bottom row. A), D) The error in the estimated mean of the prior, over the course of the experiment (each bin is 10 consecutive trials) averaged over subjects (mean +/− standard error). The far right is the average over subjects and trials. B), E) The gain, *r*, subjects used during the experiment, averaged across subjects. The bold black line indicates the Bayesian inference model's prediction for the experimental data. For comparison, the grey line is the prediction of a fixed learning rate linear filter model C), F) Inferred average prior as it evolved over the experiment.

The first 250 trials of experiment 2, before the variance of the prior switched, were identical to experiment 1. Indeed, subjects' performance during these trials was very similar (see Fig. 5). Just as in experiment 1, both groups quickly acquired the mean of the prior, with group 2A exhibiting larger uncertainty in the mean than group 2B (*p<0.001*). Subjects acquired the correct variance over a slower time-scale, statistically indistinguishable from the first 250 trials of experiment 1 (in a bin-by-bin comparison, there were no significant differences, two sample *t*-tests, *p>0.1*). The second 250 trials offered some distinctions. On average, the subject's ability to maintain a correct estimate of the prior's mean was not affected by the new variance. However, as was previously observed, the variance of the prior did influence the observed uncertainty in this estimate. For instance, with group 2A, once the prior's variance decreased to the new smaller value the variability in the average estimate decreased (see Fig. 5A, *p<0.001*). Similarly, with group 2B, after the prior's variance increased, the variability in the average estimate increased (Fig. 5D, *p<0.001*). It therefore seems reasonable that their estimate was based on the actual distribution of the prior, and not on some heuristic distinct from the prior. Furthermore, it appears that the first half of the experiment did not affect subjects' estimate of the mean in the second half of the experiment.

While learning a different prior first did not affect estimates of the mean it may have an influence on subsequent learning of the variance. Indeed, in the second 250 trials of experiment 2 there were apparent differences in the rate at which subjects learned the prior's variance. The learning rate during the second half of experiment 2 was smaller than the apparent learning rate of the first half (or equivalently, the first half of experiment 1). For example, comparing the last half of group 2A's results with the first 250 trials of group 2B (Figs 5B, E), we see that subjects in group 2A were on average slower in learning a correct estimate of the prior's variance than group 2B. Similarly, with group 2B after 500 trials the average behavior did not yet reflect an accurate estimate of the variance as it did in the first half of group 2A. Inferring the average subject prior using the measured gains and means we could track the prior as it changed over the course of learning (Figs 5C, F). It appeared as if learning the first prior somehow acted to impede subjects' ability to adapt to the second prior. This is especially evident when transitioning from a large variance to a small variance; the change in the subjects' prior was gradual (Fig. 5B).

To further analyze these results we compared them with those of an idealized Bayesian inference model. By observing the cue and target coin locations, the model accurately applied the rules of Bayesian statistics to update a joint distribution of the prior's estimated mean and variance (see Methods). The model contained four free parameters that needed to be specified before we could compare its results with those of the subjects'. Therefore, we used the data from experiment 1 to fit the model's parameters (see Methods, Appendix S1 and Fig. 3B, E) and then proceeded to compute the model's results for experiment 2. We could thus compare an ideal Bayesian model's performance with human behavior without problems of over-fitting. With more knowledge about the task than the subjects, the model represents the upper limit in accuracy on what could be observed experimentally.

A good fit to experiment 1 was obtained with the Bayesian inference model (see Fig. 3B, E, black line) by minimizing the log-likelihood of the subject data. The resulting RMS error between the subject's measured *r* values and those found with the model across both groups was 0.071. The model predicted very small errors in the prior's mean and was essentially correct in its estimate after the first few bins. This model was then used to predict the results of experiment 2. The model's behavior was qualitatively similar to the subjects' (see Fig. 5B, E, black lines). In particular note that the inference model correctly predicts no change in the prior's estimated mean, but a slower learning rate for the second half of the experiment; just as with the subject's data, the model is slow to infer the last half of experiment 2A relative to the first half of experiment 2B. Initially, the model is relatively uncertain of the prior and predisposed to estimating large changes in the variance based on the observed distribution of coins. As the experiment progresses, the model's estimate of the variance becomes more certain. By the 250^th^ trial, the model's certainty in the prior's variance makes it insensitive to the new observations of the coin's distribution, now indicating a different variance. As a result, the model is now slow to estimate the new variance, qualitatively similar to the subjects' behavior. The RMS error between the subjects' measured *r* values and those predicted with this inference model across both groups was 0.077. Again, the model's estimate of the prior's mean was essentially correct after the first few bins.

To emphasize both the goodness of fit of this Bayesian inference model, and the observed reduction in learning rate, we present a second inference model for comparison. This model, decidedly simple and non-Bayesian, only estimated the prior's variance with a constant learning rate. The model used a running window of the last ten trials to compute a sample observation of the prior's variance. The model's updated estimate was then a linear combination of its previous estimate and the current observation (see Appendix S1). Just as with the Bayesian inference model from above, this model's two parameters were fit to the data from experiment 1. Though it produced a qualitatively good fit to the data from experiment 1, this model did a poor job of predicting the second half of experiment 2 (see Fig. 5B, E, grey lines). Using the parameters obtained from the fit to experiment 1, this model predicted subjects would converge to correct estimates of the prior's variance by the end of the second 250 trials. Though this model appears to closely match the subject's behavior during the initial trials after a switch from the narrow to the wide distribution (Fig. 5E) overall the Bayesian model still performed better. The RMS error between this model and the measured *r* values across both groups was 0.205, nearly three times as large as the Bayesian inference model. This simplified inference model, with a constant learning rate, was not able to capture even the qualitative effects of experiment 2 when the prior switched from a wide to narrow distribution. Based on these results, it appears that subjects began the experiments relatively uncertain of the prior but became increasingly certain as the experiment progressed. This accounts for the apparent slower learning rate later in the experiments.

### Experiment 3

The third experiment was designed to examine a subject's ability to learn the prior's mean independent of the variance. We held the variance of the prior constant but changed its mean. At the start of the experiment, the mean of the prior was randomly chosen between one of two locations. Each subject was exposed to a minimum of 10 consecutive trials with the same prior after which there was a small probability the mean would switch to the other value on any trial. Over the course of the experiment each subject would be exposed to approximately 50 of these changes in the prior's mean. Ten subjects performed the experiment (600 trials) after being given the same instructions as in the previous experiments above (see Methods). As in the previous experiments, subjects would have to infer when the mean switched, and what the new value of this mean was in order to successfully complete the task.

**Figure 6:**
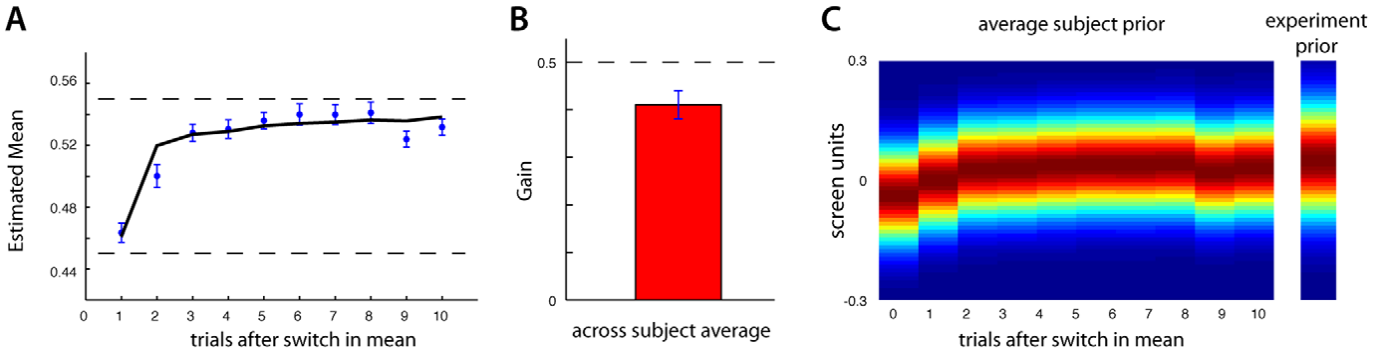
Results from experiment 3. A) The subject's estimated mean for the prior, locked on the trial the prior switched (averaged across all subjects, and all switches in the prior). The bold black line indicates the inference model's prediction of the same data. B) The subjects measured gain, *r* (averaged across the first 10 trials after a switch and across all subjects). C) Inferred average prior as it evolved once the prior switched.

Just as in the previous two experiments, subjects quickly acquired an accurate estimate of the mean even though it switched frequently. In contrast to the previous analysis, we wanted to examine the temporal aspects of this estimate on the relatively fast time scale of single trials rather than bins of 10 trials. For each subject we resorted the data, locked on the first trial after a switch in the prior's mean. This allowed us to observe the subjects estimate of the mean on a trial-by-trial basis. We then averaged theses estimates of the prior's mean across all ten subjects (see Fig. 6A). As can be seen, on average subjects were able to determine a switch in the mean almost immediately, and made changes in their strategy that reflected a correct estimate of the mean after about two trials.

To once again compare subject results with those of Bayesian statistics, we designed a new Bayesian inference model to perform the same coin catching experiment. The inference model used in experiments 1 and 2 would not be appropriate for this task. With that model's assumption of a single mean, as the experiment proceeded the model's certainty in the mean's value would grow and become progressively insensitive to new data. This decreased sensitivity to a switch in the prior's mean was not observed with the subjects, indeed, they appeared to become better able to infer the switch as the experiment progressed. With these considerations, we designed a new Bayesian inference model that had the benefit of knowing the location of the two possible means, and the correct probability of switching between these means. Therefore, to competently perform each trial the inference model needed only to estimate which of the two means was currently in use. Accurately applying the rules of Bayesian statistics, the model used the observed coin locations to estimate the correct prior. The model could then estimate the location of the target coin (see Methods). The inference model's behavior was very similar to the average subject behavior, and it's estimate of the prior's mean changed only slightly faster than the average subject behavior (R^2^ = 0.90 for the two curves, Fig. 6A). We emphasize that this Bayesian inference model had more information than the subjects and represented an upper bound on their performance.

In contrast with the previous two experiments, the average subject behavior indicated they had more difficulty in acquiring an accurate estimate of the prior's variance. Given that the two means for the prior were within one standard deviation of both the likelihood and the prior, the frequent switching may have hindered subjects' ability to accurately estimate the prior's variance. Confounding the switching of the prior's mean with the spread of coins might lead to an overestimate of the likelihood, consistent with the decreased measure of *r* observed. We averaged each subject's data over the last 200 trials to compute their estimate of the gain, *r*, and then averaged across subjects. The average *r* value was significantly smaller than the true value (*p<0.001* Fig. 6B). Using this average value and the inferred means we were able to reconstruct the subjects' average estimated prior (again, assuming an accurate estimate of the likelihood) after a switch in the experimental mean (Fig. 6C).

## Discussion

Here we have examined how the nervous system learns and represents a prior. We began our study with two hypotheses concerning learning rates – motivated by different conceptualizations of the neural coding of uncertainty. The mean and variance of a prior could be learned at the same rate, or different rates. Tracking the subject's behavior over the course of learning we were able to track how people learn priors. Our findings offer strong evidence that the mean and variance were learned accurately (given enough trials), however at different rates, and that these rates changed as the task progressed. The apparent decrease in the learning rate was consistent with a statistical model of optimal estimation that began relatively unsure, but became progressively certain of its estimates. This was contrasted with a constant-rate linear model that could not predict the change in learning rate. Moreover, our results suggest that the nervous system can efficiently learn a prior as the statistics of a task change over time, and utilize that prior in a rational manner, consistent with the predictions of a Bayesian inference model. While many studies have assumed that subjects learn priors, we have measured in detail how human subjects do so. Our results offer evidence that people can form an accurate prior, estimating its mean and variance.

This study was a first attempt to examine the temporal characteristics of how subjects acquire an estimate of the prior. To keep the scope of the paper manageable, we used a normal distribution for the experimental prior and the likelihood. Under these conditions a Bayes' optimal inference of the target coin's location is a linear function of the cue coin's location. Indeed, only two numbers were required to infer the target coin's location: an offset term and a slope (the gain *r*). The simple form of this Bayes' optimal solution confounds our ability to infer whether or not the subjects were precisely estimating the experimental prior. For instance, it could be the case that subjects do not actually learn accurate priors, but instead attempt to estimate all distributions as Gaussian, merely estimating the mean and the variance. Or in an even more uncomplicated fashion, it could be the case that subjects bypass the process of representing a prior and a likelihood altogether and merely estimate the resulting two parameters that were necessary for the optimal inference. However, we have several reasons to believe this is not the case, and subjects actually attempt to accurately represent the necessary statistics. Based on prior research, it is known that subjects react to changes in the statistics of both the prior and the likelihood. In one study in particular, when subjects were confronted with non-Gaussian distributions their behavior became predictably nonlinear [5]. This argues against a simple linear fit to the cue and target coins. Furthermore, when subjects in that study were presented with new likelihoods, they generalized in a Bayes' appropriate manner; implying subjects had learned the prior and not simply *r*. Finally, from a computational standpoint, the value *r* (which is defined with ratio of two random Gaussian variables) has a non-Gaussian distribution with a large second moment. Inferring this variable directly and accurately is therefore an unwarranted complication. Future studies could investigate the degree to which subjects accurately represent a prior and likelihood, or use approximating heuristics.

In all 3 experiments, subjects see both the cue and target coins simultaneously, on each trial. Therefore subjects can directly observe the spread of the likelihood, and receive many such observations. Samples from the prior, on the other hand, are not directly observed, but must be gathered from trial to trial. Based on these considerations, and the findings of similar work discussed above, we assumed subjects quickly estimated the likelihood of the variance, and that the dynamics of the learning process were dominated by the estimation of the prior. Assuming these considerations are valid, our study addresses the learning of priors. However, future studies could specifically examine just how quickly subjects accurately acquire an estimate of the likelihood.

Overall, subjects were able to quickly learn the mean of a prior. Relative to a Bayesian inference model, the subjects performed especially well when the mean of the prior switched repeatedly (experiment 3). The inference model, having perfect knowledge of the two possible means, merely estimated which one was currently most likely. Based on the subjects' performance, it appears they may have done the same thing; that is, rather than continuously inferring the mean based on the evidence, subjects remembered two possible means and estimated which one was being employed. It is plausible that after the subjects had been exposed to the two means repeatedly they inferred the causal structure of the task [4], [7], [16], [17]. Human behavior appears to approximate that of an optimal observer and the nervous system thus appears to use powerful learning algorithms.

There may be both statistical and neurobiological reasons for our finding that subjects took longer to learn the variance than the mean of the prior. From a statistical point of view, an accurate estimate of the variance requires more samples than that of the mean. Therefore, the relatively slow convergence to a correct variance for the prior is understandable. It could also be that subjects have real world experience that suggests phenomena change over relatively slow time scales. In addition, subjects displayed an inclination for assuming a flat prior at the outset of the experiment. Again, this could be based on real world experiences for preferring broad distributions. From a computational standpoint, it could be seen as a sensible strategy as well, neglecting a prior (or equivalently, assuming a flat prior) results in a maximum likelihood strategy (rather than a full Bayesian estimate). From a neurobiological point of view we may also expect different learning rates. Various lines of research investigating the rapid learning of a perturbation (mean) have implicated the cerebellum [18], [19], [20]. Other studies have found that uncertainty (variance) may be represented in the Basal ganglia and area LIP (e.g., [21], [22]). These areas may well exhibit slower learning than the cerebellum. The combination of these different neural structures may give rise to rapid learning of means and slower learning of variance.

We also see both statistical and neurobiological reasons for the finding that subjects were slower to adapt as the experiment progressed. The inference model, accurately applying the rules of Bayesian statistics, grew more certain of the prior as the experiment progressed. As a result, the model was slower to react to a change in the prior during the second half of the experiment. The subjects appear to be utilizing a similar strategy, perhaps growing more confident in their beliefs and less sensitive to their observations. This finding, of a change in the learning rate as the uncertainty in parameters changes is also consistent with a growing body of experimental evidence. Recent findings suggest specific cortical structures may represent a subject's level of uncertainty in task parameters [13], [14], [22], [23]. Further evidence proposes that neuromodulators may also encode uncertainty [15], [21]. These representations of uncertainty may allow the nervous system to learn relatively rapidly at the outset of the experiment, when subjects are generally uncertain of the task [24]. As the experiment progresses and subjects grow more confident in their beliefs the nervous system may learn more slowly.

In addition to the time-varying adaptation rate, it appeared as if subjects adapted asymmetrically to increases and decreases in a prior's variance, ostensibly reacting more quickly when the variance abruptly increased (Fig. 5E). Our Bayesian inference model employed a relatively simple generative model sufficient to explain the time-varying character of adaptation we focused on. Interestingly, this same asymmetric phenomena has been described in computational models of optimal Bayesian estimation of variance [25]. What's more, these same asymmetries have been observed in neural data when adapting to similar changes in the variance of a stimulus [26]. Together with our data, these theoretical and phenomenological findings offer evidence that the nervous system employs near optimal estimation of the statistics of stimuli.

Both the average subject performance and the Bayesian modeling indicated subjects quickly acquired the mean of the prior. However, in contrast with the Bayesian model, subjects continued to display relatively large amounts of variance in this estimate throughout the course of the experiments. If subjects believed the statistics of the experiment to be stationary, their variance ought to have decreased as the experiment proceeded. This was not evident. It could be that although subjects believe the environment is stationary they exhibit “non-Bayesian” behavior periodically. A well-known instance of this is the so-called matching behavior [27]. In our experiment, subjects could be exhibiting a similar phenomenon, making choices that are inconsistent with a maximum *a posteriori* estimate. Further, it could be that subjects don't strictly believe in stationary distributions, and instead entertain the possibility that changes may occur. This would imply their uncertainty (and resulting variance) should remain non-zero even at steady-state. This too would be consistent with their behavior.

The coin catching experiment is a task requiring both an inference (where is the target coin?), and a subsequent decision based on this inference (where should the net be placed?). That is, strictly speaking, subjects might be weighing their decision of where to place the net against concerns other than where they believe the target most likely to lie; e.g. how much effort is takes to move the net where they believe the target will be. Theoretically, in describing subjects' behavior we must invoke a loss or value function. However, our task was designed such that there was minimal motor effort (turning the paddle wheel). As a result, our task was effectively an estimation problem and using Bayesian algorithms we modeled it as such. Moreover, subjects' behavior might depend on other factors such as visual acuity. By displaying relatively large coins with striking colors against a relatively neutral back-drop, and having subjects seated closely in front of the computer monitor, the experiment attempted to minimize this internal measurement noise relative to the experimentally controlled cue coin variance. In these ways, the task was designed to be a probe for subjects' priors and to minimize the influence of other factors.

Tracking changing priors may open the possibility for new analyses in neural representations in sensorimotor tasks. Properties such as external stimuli or motor behaviors may be far “up”, or “downstream” from their neural representations, depending on where in the brain data is recorded from. However, pairing electrophysiological studies with the analysis performed here provides a new correlate for neural data. An evolving prior, inferred from behavioral data, may offer a closer correlate to the internal representations of a task and the resulting motor outcomes. Thus this approach may offer a new way of analyzing neural representations in a wide range of perceptual, motor, and perhaps cognitive tasks.

Priors characterize the beliefs human subjects maintain and are integral to how we decide upon actions and form decisions. Some recent models of neurological diseases such as schizophrenia are now applying the quantitative strategies of Bayesian statistics [28], [29]. Using the strategies developed in this study may thus offer novel ways of analyzing the mechanisms that give rise to neurological deficits.

## Supporting Information

Appendix S1 (0.09 MB DOC)

## References

[1] Weiss Y, Simoncelli EP, Adelson EH (2002). Motion illusions as optimal percepts.. Nat Neurosci.

[2] Stocker AA, Simoncelli EP (2006). Noise characteristics and prior expectations in human visual speed perception.. Nat Neurosci.

[3] Jacobs RA (1999). Optimal integration of texture and motion cues to depth.. Vision Res.

[4] Kording KP, Beierholm U, Ma WJ, Quartz S, Tenenbaum JB (2007). Causal inference in multisensory perception.. PLoS ONE.

[5] Kording KP, Wolpert DM (2004). Bayesian integration in sensorimotor learning.. Nature.

[6] Miyazaki M, Nozaki D, Nakajima Y (2005). Testing Bayesian models of human coincidence timing.. J Neurophysiol.

[7] Hudson TE, Maloney LT, Landy MS (2007). Movement planning with probabilistic target information.. J Neurophysiol.

[8] Berniker M, Kording K (2008). Estimating the sources of motor errors for adaptation and generalization.. Nat Neurosci.

[9] Adams WJ, Graf EW, Ernst MO (2004). Experience can change the ‘light-from-above’ prior.. Nat Neurosci.

[10] Tassinari H, Hudson TE, Landy MS (2006). Combining priors and noisy visual cues in a rapid pointing task.. J Neurosci.

[11] Thoroughman KA, Shadmehr R (2000). Learning of action through adaptive combination of motor primitives.. Nature.

[12] Kawato M, Furukawa K, Suzuki R (1987). A hierarchical neural-network model for control and learning of voluntary movement.. Biol Cybern.

[13] Behrens TE, Woolrich MW, Walton ME, Rushworth MF (2007). Learning the value of information in an uncertain world.. Nat Neurosci.

[14] Kiani R, Shadlen MN (2009). Representation of confidence associated with a decision by neurons in the parietal cortex.. Science.

[15] Yu AJ, Dayan P (2005). Uncertainty, neuromodulation, and attention.. Neuron.

[16] Wei K, Kording K (2009). Relevance of error: what drives motor adaptation?. J Neurophysiol.

[17] Tenenbaum JB, Griffiths TL, Kemp C (2006). Theory-based Bayesian models of inductive learning and reasoning.. Trends Cogn Sci.

[18] Chen H, Hua SE, Smith MA, Lenz FA, Shadmehr R (2006). Effects of human cerebellar thalamus disruption on adaptive control of reaching.. Cereb Cortex.

[19] Nezafat R, Shadmehr R, Holcomb HH (2001). Long-term adaptation to dynamics of reaching movements: a PET study.. Exp Brain Res.

[20] Lewis RF, Tamargo RJ (2001). Cerebellar lesions impair context-dependent adaptation of reaching movements in primates.. Exp Brain Res.

[21] Schultz W, Preuschoff K, Camerer C, Hsu M, Fiorillo CD (2008). Explicit neural signals reflecting reward uncertainty.. Philos Trans R Soc Lond B Biol Sci.

[22] Churchland AK, Kiani R, Shadlen MN (2008). Decision-making with multiple alternatives.. Nat Neurosci.

[23] Rushworth MF, Behrens TE (2008). Choice, uncertainty and value in prefrontal and cingulate cortex.. Nat Neurosci.

[24] Burge J, Ernst MO, Banks MS (2008). The statistical determinants of adaptation rate in human reaching.. J Vis.

[25] DeWeese M, Zador A (1998). Asymmetric Dynamics in Optimal Variance Adaptation.. Neural Computation.

[26] Fairhall AL, Lewen GD, Bialek W, de Ruyter Van Steveninck RR (2001). Efficiency and ambiguity in an adaptive neural code.. Nature.

[27] Sugrue LP, Corrado GS, Newsome WT (2004). Matching behavior and the representation of value in the parietal cortex.. Science.

[28] Fletcher PC, Frith CD (2009). Perceiving is believing: a Bayesian approach to explaining the positive symptoms of schizophrenia.. Nat Rev Neurosci.

[29] Dima D, Roiser JP, Dietrich DE, Bonnemann C, Lanfermann H (2009). Understanding why patients with schizophrenia do not perceive the hollow-mask illusion using dynamic causal modelling.. Neuroimage.

