# Supplementary material for: Learning Priors for Bayesian Computations in the Nervous System
Source: PLoS One. 2010 Sep 10;5(9):e12686. doi: 10.1371/journal.pone.0012686 (PMC2937037; doi:10.1371/journal.pone.0012686)
Supplement: Appendix S1 — (0.09 MB DOC) [file pone.0012686.s001.doc]

**Appendix**

We designed a Bayesian inference model to solve the same coin catching task as the subjects in experiments 1 and 2. The observed coin positions were drawn from the same distributions as presented to the subjects. However, the model is given the additional knowledge that the correct variance of the likelihood was $s_{l}^{2}$. On each trail, the model uses the observed target coin’s location to perform a Bayesian update of the prior’s mean and variance, *μ_p_* and $s_{p}^{2}$. Since the variance is limited to positive values a Gaussian distribution for this parameter would not be appropriate. Instead, a common choice for modeling such a joint distribution is the Normal-scaled Inverse Gamma (NIG) distribution[[1]](#footnote-2). Such a distribution assumes the mean is a Gaussian variable, and the variance has an Inverse Gamma distribution,

where -∞ < *m* < ∞, and *V*, *a* and *b* > 0. With this distribution, the expected value of the mean is

and the expected value of the variance is,

The likelihood for an observed target coin, the distribution of target coin locations, is Gaussian, and of the form,

Since the NIG distribution is a conjugate prior for this Gaussian likelihood, after observing the target coin, the model can easily perform a Bayesian update of the posterior distribution over *μ_p_* and $s_{p}^{2}$. This posterior is also a NIG distribution with the parameters updated as follows (variables with a ^+^ indicate updated vales),

Using this formalism, the Bayesian inference model is able to perform the same experiment the subjects did, using the current estimate of the mean and the variance to compute an *r* value () and then make a Bayes’ optimal prediction for the target coin’s location based on the current cue coin. After the target coin is displayed the model uses the actual target coin’s location to update the posterior distribution over *μ_p_* and $s_{p}^{2}$. This posterior then becomes the prior for the next step. The only free parameters for the model are the initial values of *m*, *V*, *a*, and *b* (a Gaussian prior would require the same number of parameters).

Since the locations of the cue and target coins could all be computed before an experiment began, many potential experiments could be obtained ahead of time. We computed the locations of cue and target coins for 1,000 potential experiments for each group (1A, 1B, 2A and 2B). Using this data, we could evaluate the model’s average performance for a given set of model parameters (*m*, *V*, *a* and *b*), and the corresponding likelihood of observing the average subject behavior on any given trial. To obtain a “best” set of model parameters, we found those values that maximized the log likelihood of the across subject binned averages of *μ_p_* and *r* for the two groups of experiment 1 (the data displayed in Fig 3). This was done numerically using custom code written for Matlab. These maximizing values, *m^*^*, *V^*^*, *a^*^* and *b^*^*, were then used to predict the average subject results of experiment 2 (the data displayed in Fig 5).

For comparison we also simulated the performance of a simple, non-Bayesian inference model that knew the true mean but estimated the prior’s variance. On each trail, the model used the observed target coins from the previous ten trials to compute a sample standard deviation, σ*_obs_*. The observed standard deviation was used to perform an update of the prior’s estimated standard deviation, $\hat{s}_{p}$ using a fixed time constant, τ.

With this simpler, non-Bayesian model there were 2 free parameters, the initial estimate of $\hat{s}_{p}$, and the gain, τ. Just as above, these two parameters were found by minimizing the difference between the average subject performance, and the model’s performance averaged over the 1,000 potential experiments for conditions 1A and 1B. However, with this model the two parameter values were found by minimizing the root mean squared error between its average *r* value for each bin and the across subject averages for each bin (where a bin was 10 consecutive trials). Minimizing the mean squared error is often equivalent to maximizing the log likelihood when assuming a Gaussian observation model. The model was then used to predict the results of experiment 2.

The NIG model we used to examine experiments 1 and 2 assumed a single fixed mean. As a result, every observation of a target coin reduced the model’s uncertainty in the mean’s value, and made a new, different mean, less likely. During experiment 3, subjects were exposed to a distribution of target coins whose mean changed approximately 50 times during the 600 trials. Therefore, to model the results of experiment 3 we designed a new Bayesian inference model, but one that inferred only the current prior’s mean. We assumed this model knew the correct variance of the prior and the likelihood. We further assumed the model knew the two possible means and the probability of switching between the two. As such, on each trial the model merely had to predict the probability that either mean was currently in use, and make the corresponding estimate of the target coin. Before any coins are observed, the agent updates the probability of the two means based on the known transition probability. Then, after having observed the cue coin in location, *x_c_*, the probability of the target coin’s location conditioned on the first prior’s mean, $m_{1}$ is,

where,

The probability of the target coins location based on the second prior’s mean is computed similarly. The target coin’s location is then computed as the expected value conditioned on the probability of the two means. Assuming *p_1_* is the probability that the current prior’s mean is $m_{1}$, and *p_2_* is the corresponding probability for the other mean, the expected value is,

After having observed the cue coin, the posterior over the two means, , is computed and used to initiate the next trial’s inference. To compare the agent’s predictions with the subjects, 10,000 simulated experiments were performed. The data for each simulated experiment was analyzed just as with the subjects, and the results were averaged.

1. See, for example, Dominici, F., Parmigianiani, G. and Clyde, M., “Conjugate analysis of multivariate normal data with incomplete observations”, *The Canadian Journal of Statistics*, Vol. 28, No. 3, 2000. [↑](#footnote-ref-2)
